# Meteorological Table
*The Observations are made at eight o'clock in the morning, two in the afternoon, and eleven at night.


**Published:** 1810-11

**Authors:** 


### METEOROLOGICAL TABLE.*

*From Oct. the 1st, to the 28th.*

| D | Therm. | Barom. | Hygrom. dry | Hygrom. damp. | Weather. |
|---|---|---|---|---|---|
| 1 | 58.64.57. | $30^{1}$ ——.-$30^{2}$ | —— | ——9 | clear . . |
| 2 | 58.63.58. | $30^{2}$ —— | —— | ——9 | clear . . . |
| 3 | 59.64.57. | $30^{2}$ —— | —— | ——9 | clear . |
| 4 | 57.64 57. | $30^{2}$ —— | —— | 11——15 | clear . . |
| 5 | 57.64.57. | $30^{1}$ ——30 | —— | ——15 | clear . . light rain in night |
| 6 | 56.61.57. | $29^{9}$ —— | —— | 20——25 | clear . . . . |
| 7 | 54.63.57. | $29^{9}$ —— | —10— | 27—— | clear . . foggy . . clear . . |
| 8 | 57.63.59. | $29^{9}$ —— | 11—13 | —— | clear . dry fog . . clear . . |
| 9 | 58.61.57. | $29^{8}$ —— | 5.10.14 | —— | cloudy . . |
| 10 | 59.60.59. | $29^{8}$ —— | 15.17.16 | —— | cloudy . . . |
| 11 | 58.60.53. | $29^{9}$ —— | 19.29.20 | —— | clear . . |
| 12 | 52.57.48. | $29^{8}$ —— | —— | 23—— | clear . . |
| 13 | 45.49.47. | $29^{9}$.30.$30^{1}$ | ——10.2 | 5—— | clear . . . |
| 14 | 47.55.56. | $30^{2}$.—— | —5— | 5—— | cloudy . ——— clear . . |
| 15 | 49.54.56. | $30^{1}$.30.—— | —— | 1—— | clear . ——— cloudy . . |
| 16 | 49.54.56. | $29^{9}$.$29^{8}$.$29^{7}$ | —— | 8.20. 10 | clear . cloudy . . . light rain . |
| 17 | 57.61.59. | $29^{6}$.$29^{6}$.$29^{4}$ | —— | 10. 3. 30 | cloudy . . . rain . . rain . . . |
| 18 | 56.62.55. | $29^{4}$.$29^{5}$.$29^{6}$ | —— | 20 — 30 | Rain . . clear . rain . . |
| 19 | 53.63.58. | $29^{8}$.$29^{7}$.— | —— | 30 — — | Rain . . cloudy . . . rain . *† |
| 20 | 59.63.58. | $29^{7}$.$29^{6}$.— | —— | 19.25. 20 | clear . rain . rain . . . |
| 21 | 54.61.63. | $29^{6}$.$29^{5}$.— | ——9 | 30.—— | cloudy . . . . rain . . rain . . . |
| 22 | 58.57.55. | $29^{3}$.——$29^{5}$ | 15.5.—— | —— | clear . rain . . clear . . *†† |
| 23 | 50.55.49. | $29^{6}$.——$29^{5}$ | —25.15. | —— | clear . . cloudy . . clear . . |
| 24 | 47.50.46. | $29^{7}$.$29^{8}$ 30. | 10.25.—— | —— | cloudy . . clear . . clear . . . . |
| 25 | 44.50.45. | $30^{1}$.$30^{2}$.$30^{3}$ | 15.25.17 | —— | clear . . . . — . . — . . . . |
| 26 | 44.50.46. | $30^{3}$.—— | 12.26.11 | —— | clear . . . . cloudy . . — |
| 27 | 47.49.43. | $30^{2}$30. $29^{9}$. | 25.15—— | —— | cloudy . . . — clear . . |
| 28 | 46.49.43. | $29^{7}$$29^{5}$. .— | —— | 20.25.—— | rain . . . rain . . cloudy . . |

* The Observations are made at eight o'clock in the morning, two in the afternoon, and eleven at night.

† Day of the month.

*† Brisk gale of wind.

*†† The process of evaporation going on with remarkable rapidity, with a strong wind at S.W. The effects of the stormy showers of the early part of the day, are not seen in the afternoon.

*Prince's-Street, Cavendish-Square.*

